# Emergence of form in embryogenesis

**DOI:** 10.1098/rsif.2018.0454

**Published:** 2018-11-14

**Authors:** Murat Erkurt

**Affiliations:** Department of Mathematics, Centre for Complexity Science, Imperial College London, London SW7 2AZ, UK

**Keywords:** morphogenesis, reaction–diffusion, bodyplan, self-organization, genetic regulatory network

## Abstract

The development of form in an embryo is the result of a series of topological and informational symmetry breakings. We introduce the vector–reaction–diffusion–drift (VRDD) system where the limit cycle of spatial dynamics is morphogen concentrations with Dirac delta-type distributions. This is fundamentally different from the Turing reaction–diffusion system, as VRDD generates system-wide broken symmetry. We developed ‘fundamental forms’ from spherical blastula with a single organizing axis (rotational symmetry), double axis (mirror symmetry) and triple axis (no symmetry operator in three dimensions). We then introduced dynamics for cell differentiation, where genetic regulatory states are modelled as a finite-state machine (FSM). The state switching of an FSM is based on local morphogen concentrations as epigenetic information that changes dynamically. We grow complicated forms hierarchically in spatial subdomains using the FSM model coupled with the VRDD system. Using our integrated simulation model with four layers (topological, physical, chemical and regulatory), we generated life-like forms such as hydra. Genotype–phenotype mapping was investigated with continuous and jump mutations. Our study can have applications in morphogenetic engineering, soft robotics and biomimetic design.

## 1. Background

The morphogenesis of an embryo is the emergence of its form from the singularity of a zygote. The theory of embryonic development has been a long-standing issue in biology and can be traced back to Aristotle. In Book II of *On the Generation of Animals*, Aristotle rejects preformationism and introduces the concept of epigenesis and orderly development towards a final cause. Meckel and Serres, in the eighteenth century, proposed the recapitulation theory. The embryogenesis of the higher-order organisms was a progression through the formation stages of lower life forms. In the nineteenth century, Haeckel expanded recapitulation to biogenetic law. An animal embryo’s development was the replay of its species evolutionary forms; ontogeny recapitulated phylogeny. Later, Roux introduced the mosaic theory. Embryonic development is a well-choreographed unfolding of form; after a few divisions of the zygote, the cells attained a fate that would generate specific parts of the developing organism. With the advances in genetics, the debate of ‘neo’-preformationism and epigenetics intensified. Currently, we seek a theory of embryogenesis that would explain the unfolding of form from the singularity of a zygote.

Pattern formation is a process of building spatial structures out of none. In his seminal paper ‘The chemical basis of morphogenesis’, published in 1952 [1], Alan Turing argued that a nonlinear dynamical system of the two reactants diffusing on a tissue at different rates can spontaneously generate a pattern starting from a homogeneous initial condition if provided with some perturbation. Gierer & Meinhardt [2] published their equivalent of reaction–diffusion formulation for pattern formation, which was based on interacting morphogens that had short-range activation and long-range inhibition, very much similar to the morphogen reactants proposed by Turing. However, their theory was further expanded with morphogen sources that had shallow gradients across the tissue. Such source gradients were not generated by the morphogen dynamics but were driving it exogenously. Gierer and Meinhardt claimed that the source densities were expected to change slowly as an effect of cell differentiation. While the proposed theory could explain the generation of morphogen peaks or spatial periodicity, it did not explain how the driving shallow source gradients were formed in the first place. Since then, various types of reaction–diffusion models have been introduced [3]. Although mathematically elaborate, reaction–diffusion as a mechanism for pattern generation in animals is criticized due to its need for the tuning of system parameters, limited integration with tissue mechanics and scarce *in vivo* evidence.

Although Turing’s reaction–diffusion system constitutes an important framework for explaining self-organization through local interactions, it generates patterns, not form. As Turing allegedly said [4] regarding the formation of zebra skin patterns:

Well, stripes are easy, but what about the horse part?

The generation of form requires spatial symmetry breaking at the organism length scale, whereas pattern formation is based on breaking the local symmetry, which results in spatial quasi-periodicity. Form generation in a living object, starting with the establishment of a bodyplan, requires cells to know their positions. This is the basis of positional information theory that was developed by Wolpert [5]. Cells are assumed to acquire positional identities through a coordinate system formed by morphogen gradients that span the organism or its subregion. Subsequently, they differentiate based on such positional information.

In stark difference to Turing’s reaction–diffusion systems, the positional information theory was built on *in vivo* experimental results rather than mathematics. Morphogen gradients that provide positional information are observed in many developing systems [6]. Early studies on chick, hydra and insect limbs showed the existence of morphogen gradients along the main body axes [7,8]. Recent experimental data show that Dpp, Sonic hedgehog (Shh) and activin exhibit gradient distribution across the organism during embryonic development [9]. Such gradient thresholds trigger the expression of target genes [10]. Other studies showed that the hedgehog family of proteins have spatial gradients on *Drosophila*’s larval cuticle and wing imaginal disc, as well as on the vertebrate neural tube [11]. In *Xenopus* embryo, activin gradients correlate with the expression of *goosecoid* and *Xbra*.

In the vertebrate neural tube, Shh protein acts as the gradient-forming morphogen and drives the spatial patterns of gene expression, resulting in subdivision of the ventral neuroepithelium into five domains with distinct neuronal subtypes [12,13]. Recent *in vivo* studies, supported by mathematical modelling of nonlinear dynamics, has shown that the Shh morphogen gradient is interpreted by the downstream genetic regulatory network (GRN) by changing the intracellular Gli activity that triggers the transcriptional circuit, resulting in generation of spatial patterns of gene expression [14].

Despite this evidence of gradient distribution and corresponding gene activation, there are limited theories on the way such axis-defining gradients are formed and converted into precise positional information [15]. Possible mechanisms such as self-enhanced morphogen degradation and competition between the morphogens for binding to inhibitors have been suggested [16]. Although the pre-patterning of an organizer is assumed, it is not clear how such an organizer would establish a smooth gradient distribution across the tissue. Typically, such gradients are based on secreted molecules that can diffuse extracellularly. Alternative transport mechanisms based on cell-to-cell transport have been proposed [17,18]. Moreover, it is challenging to maintain the established gradient against noise, degradation and fluctuations at the organizer. Any acceptable model must ensure reproducibility and robustness [19,20,21].

Turing’s reaction–diffusion theory and Wolpert’s positional information theory were perceived as opposing camps in explaining morphogenesis [22]:

This [positional information-based] view of pattern formation must be contrasted with those views which explicitly or implicitly claim that in order to make a pattern it is necessary to generate a spatial variation in something which resembles in some way the pattern … View of pattern formation is characterized by the work Turing [1] and is the antithesis of positional information.

Meinhardt introduced exogenous source gradients into his equations, which provided positional information to the otherwise isotropic reaction–diffusion system [23]. This was the first step towards amalgamating reaction–diffusion with positional information theory. However, attempts to establish positional information through reaction–diffusion systems faced difficulties such as (i) the generation of secondary or periodic peaks as tissue size grows, (ii) exponential decay of gradients, (iii) difficulty in establishing orthogonal gradients for primary and secondary body axes, and (iv) forming an exact number of segments [24]. Several elaborate remedies were proposed within the framework of the reaction–diffusion systems [25–28].

### 1.1. Need for ‘self-organized’ organizers

Typically, an early blastula is symmetrical topologically and information-wise. A complete embryo can be formed from a fragment of the early blastodisc not containing the posterior marginal zone [29]. These experimental observations indicate that the organizer regions of the tissue that determine the bodyplan of an embryo are emergent and self-organized. Despite the elegant mathematics of Turing’s reaction–diffusion systems for pattern formation, and notwithstanding the well-established experimental evidence of positional information based on gradients in the embryo, to date, there does not exist a theory that can robustly explain the self-organization of the organizers that would reproducibly and reliably generate the immense variety and detail of forms in phyla.

## 2. Theory

### 2.1. Vector–reaction–diffusion–drift

Pattern-forming reaction–diffusion systems use local kinetics and isotropic diffusion, which act as spatial wavevector-selecting operators with the generic form
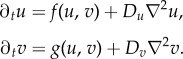
Some well-studied reaction–diffusion systems include the Gray–Scott, Barrio–Varea–Aragon–Maini and Gierer–Meinhardt models. These models generate pattern but not form. Pattern is a spatial organization that is locally differentiated but globally repeated, whereas form is a spatial organization that is globally differentiated. Generating form requires the tissue to have unique differentiation such as anterior–posterior and dorsoventral axis formation. Reaction–diffusion systems cannot generate global symmetry breaking; they act as local spatial filters that feed on random fluctuations or disturbances to generate spatial differentiation within the characteristic distance of the underlying diffusion dynamics. Form of an organism, on the other hand, is a juxtaposition of topological symmetry breakings, which can be achieved in a system that incorporates a local sense of directionality, which traditional reaction–diffusion systems lack. In the Gierer–Meinhardt model, spatial isotropy is extrinsically broken by the introduction of exogenous source densities.

Our vector–reaction–diffusion–drift (VRDD) system creates self-organized tissue-wide symmetry breaking and, thus, is capable of generating form. It has three building blocks: (i) self-organization through percolation of locally generated information (*source–field coupling*); (ii) local directionality introduced as drift on field gradients (*symmetry breaking by drift*); and (iii) topology encoded in the interaction matrix of the morphogen vector, which sets the organizing axes (*pillars of form*).

### 2.2. Source–field coupling

The *source–field* concept is introduced herein where *source* is the ‘organizer’ in Wolpert’s nomenclature of positional information theory [30] and *field* corresponds to the ‘morphogenetic field’. *Source–field* coupling is defined by equation (2.1). Any change in *source* ripples through the *field* based on the spatio-temporal solution,2.1
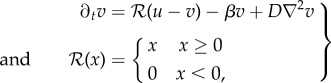
where 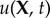 is the *source*, 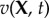 is the *field* and 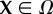 is the tissue. 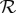 is the ramp function though which *source* pumps up the *field*. *β* and *D* are the degradation and diffusion rates of the *field*, respectively. In the proposed model, *field* is generated by the localized *source* and dispersed through the tissue by free extracellular diffusion.

### 2.3. Symmetry breaking by drift

The concept is motivated by the following ‘Lego game’ problem that we pose:

A group of *N* players join in a circle; each player is given a bag of a random number of Lego bricks. Each member can exchange Lego bricks with members on their left or right in the circle. Members are undifferentiated, and they all execute the same rule set. The goal is to combine all Lego bricks at one member.

**Figure 1.**
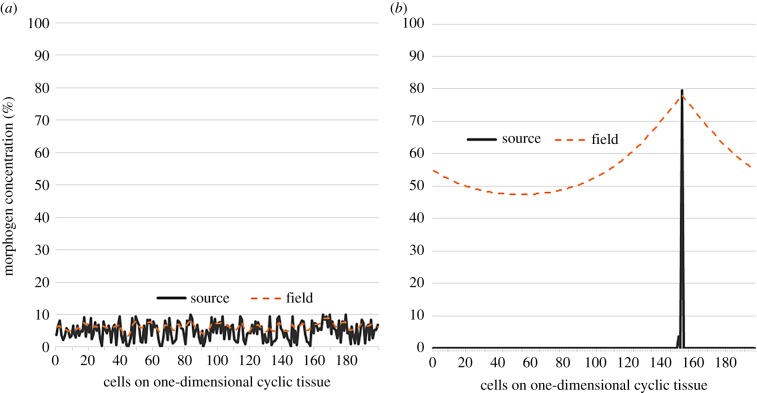
Initial versus limit-cycle distribution. One-dimensional cyclic tissue with VRDD dynamics consisting of 200 cells. (*a*) The initial condition with each cell having a random (noise level) morphogen *source* concentration. (*b*) The steady-state attractor of the system. After *ca* 100 steps, the *source* concentration falls to zero in all cells, except the organizer cell that manages to aggregate all morphogen sources. It is important to note that the organizer cell was not preset exogenously but emerged as self-organization of this nonlinear spatially distributed dynamical system. The dashed line is the corresponding steady-state morphogen *field* distribution. Parameters used: *w* =−0.1, *D*_*u*_ = 0, *β* = 0, *D*_*v*_ = 0.2. (Online version in colour.)

Such a system, starting from a random initial condition with no spatial correlation, ending in a unique spatial distribution of Dirac delta-type function, would be achieving global symmetry breaking with local interactions. We propose that such a system can be implemented by a drift of *source* on the gradient of *field*. The drift of a morphogen *source* *u* based on a generic field **F** is given by 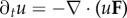. Hence, our *source–field* coupled reaction–diffusion–drift system is given in equation (2.2),2.2*a*
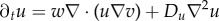
and2.2*b*
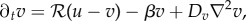
where 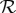 is the ramp function and *w* is the drift rate (positive as attraction, negative as repulsion). The limit cycle of this spatially distributed dynamic system is a Delta dirac-type singular peak with a system-wide broken symmetry. Figure 1 shows the initial and final condition of the *source* and *field* distribution on a one-dimensional (1D) cyclic domain running equation (2.2).

### 2.4. Pillars of form

We expand the Lego game into multi-colour. The goal is to have pillars of the same colour with specified relative positioning to other colours. This is the conceptual framework for our morphogenetic vector model. The VRDD system is based on an interacting network of *M* morphogens of *source–field* pairs. Each morphogen *source* reacts to others through a drift on their gradient fields with weights set by the interaction matrix **W**. The vector formulation of the VRDD system is given in equation (2.3),
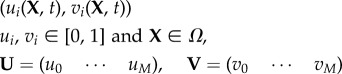
2.3*a*
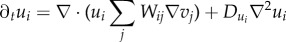
and2.3*b*
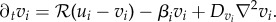


VRDD is a spatially distributed nonlinear dynamical system where the limit-cycle attractors are Dirac delta distribution of *source* types with relative positioning set by the interaction matrix.

Let us consider a VRDD system run on a spheric surface with two morphogens (R, G); and the interaction matrix is set as 
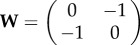
. Then the R *source* will be drifting away from the G *source* based on the gradient of the G *field* (because 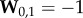), and similarly the G *source* will be drifting away from the R *source* based on the gradient of the R *field* (because 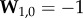). The steady-state spatial configuration will be R and G *sources* concentrated as polar opposite peaks on the sphere, forming an anterior–posterior body axis of the spheric tissue in a self-organized manner.

Now let us consider the case where the VRDD system is again run on a spheric surface with four morphogens (R, G, B and Y), and the interaction matrix is set as 
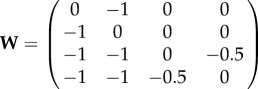
.

R and G will be again drifting away from each other based on each other’s *field* gradient; and they would be ambivalent to *fields* of B and Y (because 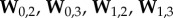 are zero), aligning themselves as two polar opposites similar to the previous case. B and Y, on the other hand, will be drifting away from both R and G (because 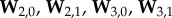 are −1), hence they would be pushed to the equator of the R, G poles. Secondarily, B and Y are being pushed away from each other (because 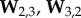 are −0.5), aligning as polar opposites on the equator of R, G. The steady-state spatial configuration will be the dual body axis (anterior–posterior and dorsoventralis) formed by the RG and BY poles, respectively.

### 2.5. Biological implications of vector–reaction–diffusion–drift

Here, we discuss the biological implications of our VRDD theory on its three building blocks. We conceptualize the Shh and Bicoid (Bcd) proteins and their encoding genes as the basis for biological relevance to our VRDD model. Shh is a well-studied signalling molecule in development that has a crucial role in embryogenesis [11]. Shh is known to be generated in organizing regions and to form long-range gradients that act as positional information for development. For example, the notochord found ventral to the neural tube (transient during vertebrate development) plays a key role in signalling and coordinating development during gastrulation and provides the organizing region for Shh generation which subsequently diffuses to generate the morphogen gradient. Also, in early tooth development, the region called the primary enamel knot generates Shh, which then leads to the morphogen gradient that provides positional information. Similarly, Bcd protein and its encoding gene has been widely studied as a morphogen gradient [31] and is relevant for conceptualizing our VRDD theory’s biological implications.

(i) *Source–field coupling*. Our VRDD model would predict the existence of an activator of Shh gene expression which would correspond to the *source*. High concentrations of *source*, i.e. a high concentration of the Shh gene activator, would drive the level of Shh protein expression. The *field* would be the Shh protein concentration itself. The ramp function in equation (2.1) essentially says that the *source* drives field generation until the *field* concentration exceeds the *source* concentration. Therefore, our VRDD model would predict the existence of an inhibitor of Shh gene expression, which would be triggered once the field concentration exceeds that of the source. This catalytic system would effectively generate the source–field coupling. The numerical solution to equation (2.1) shows a rapid decay close to the *source*, but a more gradual linear-like decline away from the *source*, which has been a proposed requisite for morphogen-based positional information to have robustness and long-range action [20].
(ii) *Symmetry breaking by drift*. The key concept of our VRDD model is organism-wide topological symmetry breaking achieved by local gradient-based drift (hence the name reaction–diffusion–drift model). In its continuous-space formulation, drift requires a mechanism that would generate directional diffusion based on the direction of an underlying gradient field. Such directional diffusion is difficult to generate in a chemical system. However, once we consider discretizing our equations in space as provided in §3.5, the results lead naturally to a cell-to-cell transport-based mechanism. In discretized formulation, the field gradient operator becomes a difference of concentration across the set of neighbouring cells of a given cell. Hence, gradient-based drift is an active transport of *source* based on *field* concentration differences of neighbouring cells. This can be biologically realized by a juxtacrine signalling-based mechanism. Our VRDD model would predict that the ligand–receptor concentration on the boundary of two cells on such a juxtacrine signalling system should be proportional to the *field* difference between such cells. Hence, higher differential cell pairs would have higher transport, resulting in discretized gradient drift. Such discretized models based on cell-to-cell signalling for morphogen transport and pattern formation have been proposed [32,33].
(iii) *Interaction matrix of the morphogen vector*. The morphogen vector in our VRDD model consists of a set of morphogens with their respective gradients. The drift is based on the perceived single field, which is a linear combination of the fields of the morphogen vector, which is defined by the corresponding row of the interaction matrix. To biologically realize such a linear combination, our VRDD model would predict that a kernel of activators (or inhibitors) should exist that would drive the ligand–receptor concentration on the cell boundaries based on their corresponding field differences of each morphogen. The weights in this kernel would define the interaction matrix row components. Such a system would generate equation (2.3) of VRDD model.

### 2.6. Genetic regulatory network as a finite-state machine

Various theoretical models for cell differentiation using biochemical networks have been proposed. The ‘threshold Boolean network’ model was introduced by Kauffman [34], and has since been used extensively for modelling the GRN dynamics, including the fission yeast cell cycle, *Arabidopsis thaliana* floral morphogenesis and the mammalian cell cycle [35]. In these models, differentiated cell types correspond to attractors of the underlying nonlinear dynamical system. The globally coupled metabolic reaction network with active transport of chemicals has been shown to generate clustering of differentiated cells [36]. Neural-network-type models were also considered [37,38]. Spatially extended GRN models have been recently used for pattern generation [39]. A network of morphogens reacting through a neural-network-type function where spatial connectivity is either a traditional diffusion or direct contact induction (effectively a discretized cellular model) has categorical pattern-generating capabilities [40].

Complex pattern formations do not need to be due to complicated hierarchical and layered GRNs. Cells that have the same genome can differentiate into clusters of different behaviour based on their developmental history, indicating that epigenetic lineage is likely to be switching the genetic stage of a cell [41]. Spatial complexity emerges when epigenetic information provides feedback to the GRN. Developmental mechanisms that have modular reuse of the genetic tool-set, coupled with epigenetic feedback, can generate a large variation of adaptive morphologies and shall be selected by evolution [40].

We introduce dynamics for cell differentiation where a genetic regulatory network is modelled as a finite-state machine (FSM). An FSM is an abstract mathematical model of computation where the machine can be in one of a finite number of states at any given time and transitions from that state in response to external inputs.

**Definition 2.1.**

An FSM is defined by a 5-tuple {*Q*, *q*_0_, *X*, *Σ*, *δ*}, where

— *Q* is the set of FSM states
— *q*_0_ ∈ *Q* is the start state
— *X*⊆ *Q* is the set of final states
— *Σ* is the set of symbols representing input to the FSM
— *δ* : *Q* × *Σ* is the transition function of the FSM.

**Figure 2.**
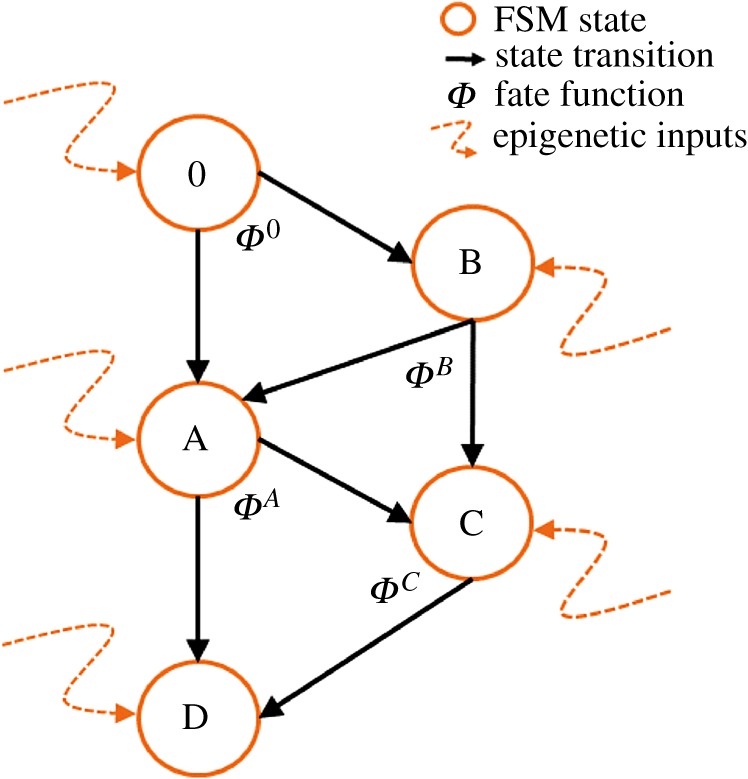
FSM model of a genetic regulatory network. Each cell can be at one of a finite number of states, defined by its gene expressions, at any given time. The cell can change its state and transition to another state based on its fate function. Fate function is a function of epigenetic information that the cell has at any given time such as morphogen concentration levels at the cell location. Embryonic stem cells start from state 0. Each cell state is a differentiation. If a cell is reset to state 0, it will start acting as a stem cell. (Online version in colour.)

As depicted in figure 2, the FSM can be represented as a directed graph where each node constitutes a state of the machine, and the edges depict the possible transition from such a state to the next connected state. In this representation, the FSM’s transition function *δ* is the set of fate functions *Φ*_*q*_ for each state. The outgoing edge of a node is selected based on its fate function and external inputs to the FSM at any given time.

In our FSM model of a genetic regulatory network, each cell state is defined by the expression of underlying genes. The input set for fate functions is dynamically changing epigenetic information consisting of local morphogen concentrations plus the ‘state timer’, which is a time-decaying local morphogen that gets reset upon state transition. The state-timer morphogen provides time dimensionality to switching logic of the FSM. It is a crucial part of FSM design through which stasis and ageing are introduced to morphogenesis. Without losing generality, the FSM can be reduced to a situation where each state has two connections, and, therefore, fate functions become a tripartite complete and non-overlapping partitioning of the input set **Σ** into **Σ**_0_, **Σ**_1_, **Σ**_2_. Let 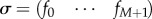 represent the vector of local morphogen concentrations, including the state timer. The fate function is given as
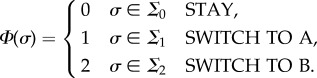
We can further reduce fate function *Φ* so that the tripartition of the input space can be scripted as a logic function. Let *φ*_*α*_ denote a threshold gate
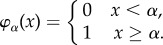
Let 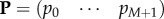 denote the logic values of input set *σ*, where 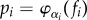, and let 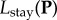 and 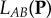 be logic functions on **P**. Then, the fate function becomes2.4
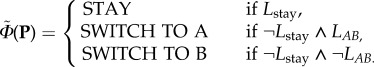

**Definition 2.2.**

A reduced fate function 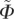 is defined by a list of threshold values and two logic functions {*α*_0_, …, *α*_*M*+1_, *L*_stay_, *L*_*AB*_}.

**Definition 2.3.**

A GRN state *S* is defined by its reduced fate function and two connected ‘next’ states 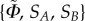.

In this model, the GRN of the zygote is at the start state of the FSM. All other states of the FSM, by construct, are reachable through a set of state transitions. As cells divide, each new cell begins to run its own FSM, commencing from the state of the parent cell. Cells in the start state are omnipotent cells; they can transition into any further cell type given a particular sequence of external inputs triggering state transitions.

The inputs to fate functions are the local morphogen concentrations that develop over time through the underlying VRDD systems. This model allows us to generate spatial subregions consisting of cell neighbourhoods with common FSM states and grow complicated forms hierarchically in spatial subdomains. Cells that are at a given FSM state have common gene expressions that determine the functional parameters of the cell; in our case, these are the parameters of underlying VRDD systems, including the interaction matrix **W** and the growth kernel **g**.

Our FSM model allows morphogen gradients to be re-established in smaller subregions in a dynamical fashion, which allows for hierarchical form generation. Dynamically changing morphogen gradients, rather than a static one, have been experimentally observed and been proposed as a dynamical system-based generalization to Wolpert’s static positional information [42]. Our FSM model incorporates such a dynamically (and subspatially) changing gradient concept.

## 3. Model

Integration of reaction–diffusion dynamics to tissue mechanics requires a chemical state to drive cell physics such as growth, division, motion, adhesion and apostosis; and the mechnanical state, such as convexity, deformation, compression to provide reverse cues to chemical reactions [43]. Coupling tissue mechanics with morphogen dynamics can result in robust pattern generation [44]. In the last decade, simulation environments that model tissue mechanics and chemical dynamics have been introduced, such as MecaGen [45], SynBioTIC [46] and COMPUCELL [47,48].

We developed an integrated simulation model to demonstrate self-organization of form in embryonic development starting from the blastula stage and advancing to gastrulation using the VRDD and FSM concepts outlined above. The term blastula stage refers to the period between 128 cells to *ca* 10 000 cells (14th zygotic cell cycle), where the early embryo is a sphere of blastomeres that surround the fluid-filled inner cavity. During this stage of development, a significant amount of activity occurs to establish cell polarity, cell specification and axis formation. After the blastula stage, gastrulation commences where the spheric topology starts to change under the impact of organizing axes. This is the onset of morphogenesis in the embryo. As Wolpert noted [49]:

It is not birth, marriage, or death, but gastrulation which is truly the most important time in your life.

Our simulation model consists of four layers: topological, physical, chemical and regulatory, with causation starting from the regulatory layer, cascading down to the topology layer. There is feedback from the chemical layer to the regulatory layer, governed by an FSM model. In the current version of the model, there is no feedback from physical or topology layers upwards.

(i) *Topology layer*. Blastula-stage embryo is modelled as a spherical mesh with each vertex representing a cell. This layer sets the spatial form of the organism and manages cell-to-cell connectivity, cell growth and cell splitting.
(ii) *Physical layer*. Tissue mechanics of the embryo, including differential growth and elasticity, run at this layer with minimally descriptive physics capturing the essential dynamics.
(iii) *Chemical layer*. VRDD system is simulated at this layer. Morphogen levels act as cues that feed downwards to the physical layer, generating differential growth. Morphogen levels also act as cues that feed upwards to the regulatory layer, determining state switching of the FSM.
(iv) *Regulatory layer*. FSM model of the genetic regulatory network is maintained in this layer.

### 3.1. Topology layer

The topology layer constitutes a mesh graph where each cell of the embryo is a vertex of the graph, and cell-to-cell contact is represented by edges between the nodes. The form of the embryo is represented as embedding of the mesh graph in *R*^3^. Each vertex has a position vector 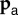, and each 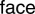 has a unit normal vector 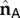 (defined as pointing away from the organism). We use the following notation.

**Definition 3.1.**

**G(V, F, E)** denotes the mesh graph of the blastula, where vertices are 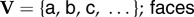 are 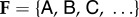; and edges are 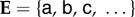. 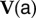 denotes the set of vertices of *edge a*; 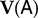 denotes the set of 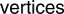 of 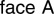; and 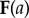 denotes the 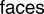 of *edge a*. Additionally, let 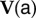 denote the set of first-degree neighbour vertices of vertex a, which is defined as having a common edge; i.e. 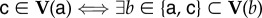. For the triangular closed-surface mesh topology, we have the following cardinality rules: 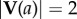, 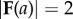, 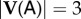.

We used the so-called half-edge data structure to represent the mesh graph, where each edge of the graph is composed of two oppositely directed half edges. This data structure puts connectivity information of the mesh into half edges, instead of faces. The half-edge data structure is preferred, as it minimizes the amount of data storage and allows for an effective way of modelling cell splits in the embryo through edge splitting. Our data structure was built in C++; no external mesh library was used in this project. A key feature of the topology layer implementation is dynamical cell splitting. As nodes change their spatial position during differential growth, edges get stretched. Cell splitting is implemented by dividing edges, which creates new nodes in the middle of split edges. The mesh data structure dynamically updates itself, maintaining a typical cell size as the organism grows.

### 3.2. Physical layer

The stretchiness and bendiness of the blastula is modelled by two spring types on each edge, one connecting its two nodes and the other one its two faces: the node-to-node spring with a rest length of 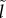, which regulates the stretchiness of cell, and the face-to-face spring with a rest angle of 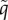, typically 0, which regulates the bendiness of the tissue. We acknowledge that the physical layer we are proposing oversimplifies the tissue mechanics of a real embryo at the gastrulation stage. Our purpose in this model is to create a simplistic topological medium that allows for the morphogen concentrations generated by the VRDD dynamics to generate differential growth. The topology of our model is a two-dimensional (2D) mesh–membrane surface, which is clearly a significant deviation from the biological tissue reality of three dimensions. Our modelling of the tissue boundary as a 2D mesh–membrane has two reasons: firstly, tissue growth arguably is predominantly on the surface, and, secondly, *in silico* implementation of a model that runs on the 2D surface rather than the three-dimensional (3D) body decreases computing implementation load (memory and processing time) by an order of magnitude 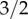 (for example, 10 000 cells on the tissue boundary would correspond to a 1 000 000 cell simulation in a 3D body).

The energy of an edge is defined as the sum of the Hooke energies of its two springs,3.1
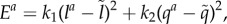
where 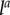 is the linear distance between the two vertices of edge *a*; 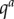 is the angular distance between the two faces of the edge *a*; and *k*_1_, *k*_2_ are the respective spring constants,3.2
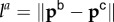
and3.3
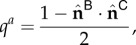
where 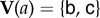 and 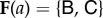. The energy of a vertex is defined as the sum of its edges’ energies,3.4
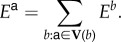
The differential growth of the tissue is modelled as a movement of the cellular vertex normal to the tissue surface governed by equation (3.5),3.5
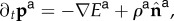
where 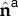 is the surface unit normal vector at the position of vertex a, defined as an average of the unit normal vectors of the faces that connect at a, which is
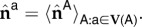
Differential growth is driven by the spatially distributed growth factor *ρ*, which is an output of the chemical layer of our model. In biology, embryonic tissue growth is driven by various mitogenic growth factors, such as insulin-like growth factors and epidermal growth factors, which, in our model, are simplified to a mitosis-inducing, spatially distributed, dynamical generated variable *ρ*, given by equation (3.7).

### 3.3. Chemical layer

At the core of form generation is the VRDD system, which is managed at the chemical layer. This section explains the manner in which the VRDD is spatially discretized and the way it runs on the mesh with each vertex representing a cell of the tissue. Each cell has *M* morphogens of the *source–field* pair. Let 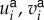 denote the *source* and *field* concentrations of morphogen type *i* at vertex location a,
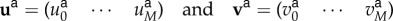
are the morphogen vectors of *source* and *field*. The VRDD system is denoted as3.6
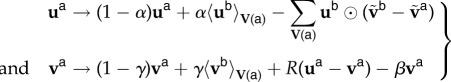
and3.7
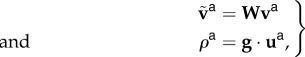
where 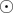 denotes element-by-element multiplication of two vectors; **W** and **g** are the interaction matrix and growth kernel, respectively. Growth factor *ρ* is the linear combination of local morphogen concentrations with weights set by the growth kernel **g**. The biological implication would be that local morphogen concentration levels act as activators or inhibitors to expresion of the growth factors, set by the weights in the kernel **g**.

### 3.4. Regulatory layer

**Definition 3.2.**

The genotype of an organism is the set of FSM states {*S*_*i*_}, where each *S*_*i*_ consists of a fate function, two connected states, an interaction matrix and the growth kernel, 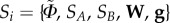.

The FSM model of the genetic regulatory network is run at the regulatory layer based on the organism’s genotype. This layer feeds state parameters to chemical and physical layers and receives back morphogen concentrations from the chemical layer as epigenetic information to be used for input to fate functions. In our current model, there is no feedback from physical or topology layers upwards to the regulatory layer. Local information such as the stretch and bend of tissue, as well as local convexity, may be used as mechanical cues to the regulatory model in future work.

### 3.5. Discretization

#### 3.5.1. Spatially distributed locally coupled maps

A map is a discrete-time dynamical system where the state of the system at the next time step is based on a map (function) of the current state. Spatially distributed locally coupled maps are extended systems that consist of a set of nodes organized on a lattice or a mesh-type graph structure. Each node runs its own map but with a state update based on the combined state of the node and its neighbours. Update timing is an important part of the description of the system; synchronous or asynchronous update schemes can also be used. The general form of a vector morphogen system with *M* types of morphogens, each consisting of its *source* (*u*) and *field* (*v*) pairs, running on discretized space and time, is as follows:
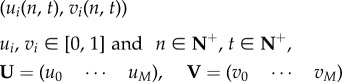
3.8*a*
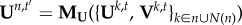
and3.8*b*
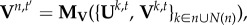
where *N*(*n*) denotes the set of neighbours of node *n* setting the spatial graph topology, and maps 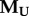 and 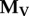 define the discrete dynamics.

#### 3.5.2. Diffusion and drift on the mesh

**Gradient operator.** The gradient on mesh space is defined for each node site *n* as the vector of the dimension equal to the cardinality of the neighbour set of *n* as follows:
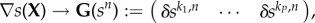
where
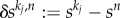
and *k*_*j*_ ∈ *N*(*n*), neighbours of node *n*.

**Divergence operator.** Divergence on the mesh space for a vector field defined between such a node *n* and its neighbours (on the edges) is given as follows:
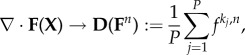
where *f*^*m*,*n*^ denotes the vector field pointing from node *n* to its neighbour node *m*. Divergence measures the net amount of vector field emanating from the node *n*.

**Laplacian operator.** Laplacian on the mesh, as the divergence of the gradient field, reduces to the deviation of node *n* from the average of its neighbours, as follows:
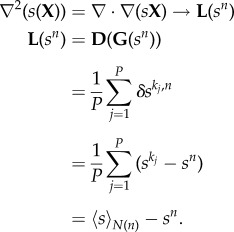


**Diffusion.** The diffusion equation discretized on a mesh becomes3.9
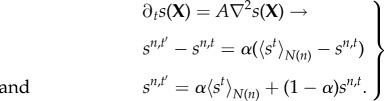
Therefore, diffusion on the mesh is equivalent to the *averaging* of a node’s state towards its neighbours’ state.

**Drift over gradient field.** The drift over the gradient field, which is at the core of the reaction–diffusion–drift formulation, becomes3.10
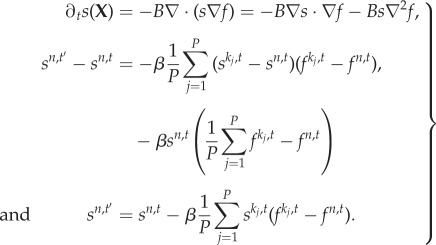
Therefore, the gradient drift on the mesh is equivalent to the field-weighted flow of neighbour states into the node.

**Vector–reaction–diffusion–drift model on the mesh.** Applying the discretization techniques outlined above, the VRDD model on a mesh is stated as3.11
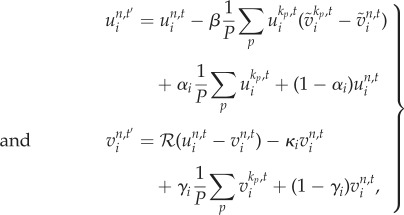
where
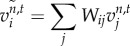
and
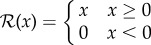
with the interpretation that *u*_*i*_ is the morphogen source of type *i*, and *v*_*i*_ is the respective morphogen field, whereas 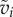 is the perceived field by morphogen source *i*, based on the attraction–repulsion relationship among the morphogen types defined by interaction matrix **W**.

## 4. Results

### 4.1. Fundamental forms

All forms that are topologically homeomorphic to a sphere can be transformed from it by a series of stretchings that are normal to the surface at the organizing pole points. With no organizers, all points on the sphere are undifferentiated and equivalent. We identified three fundamental forms based on the symmetry groups on a sphere.

(1) *Single organizer*. All points on the sphere can be grouped in terms of distance to the single organizing pole, resulting in equivalent sets of concentric rings. This is a single-axis bodyplan (anterior–posterior) with rotational symmetry.
(2) *Double organizer*. Two organizing poles (which are not trivially on the same line as the centre) subdivide the sphere into two mirror halves. Any point on the half sphere can be uniquely identified in terms of its distance to the two organizing poles. This is a dual-axis bodyplan (anterior–posterior and dorsoventral) with mirror symmetry.
(3) *Triple organizer*. The three organizing poles (which are not trivially on the same line as the centre) fully break the symmetry of the sphere, and all points can be uniquely identified in terms of distance to such three poles. This is a triple-axis bodyplan. There are no known life forms on earth with a triple-axis bodyplan.

**Figure 3.**
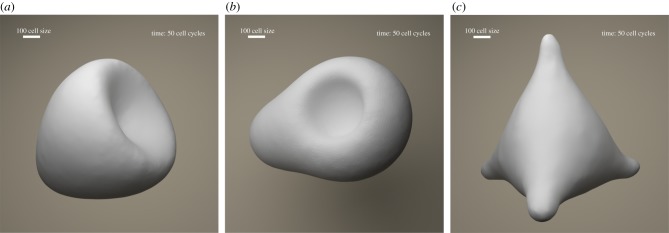
Fundamental forms. Initial conditions in all simulations are spheric blastula with *ca* 2000 cells. Gastrulation simulation time is 50 cell cycles. The resulting embryonic form has *ca* 5000 cells. Organizing polar regions are self-organized as an emergent property of the specified VRDD. (*a*) Single body axis (anterior–posterior) formed using a single state FSM and two-morphogen VRDD. (*b*) Dual body axis (anterior–posterior and dorsoventralis) formed using a single state FSM and four-morphogen VRDD. (*c*) Triple body axis is formed using a single state FSM and four-morphogen VRDD. Interaction matrices and growth kernels are as follows, respectively: 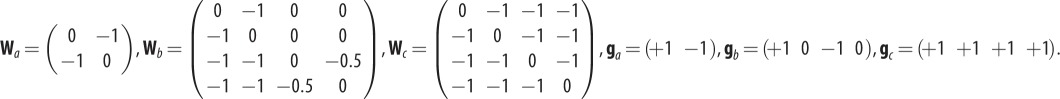 (Online version in colour.)

We generated the three fundamental forms using the VRDD system. The results are shown in figure 3. These forms are generated using a single-state FSM and are the building blocks for more complicated forms.

### 4.2. Making a hydra

**Figure 4.**
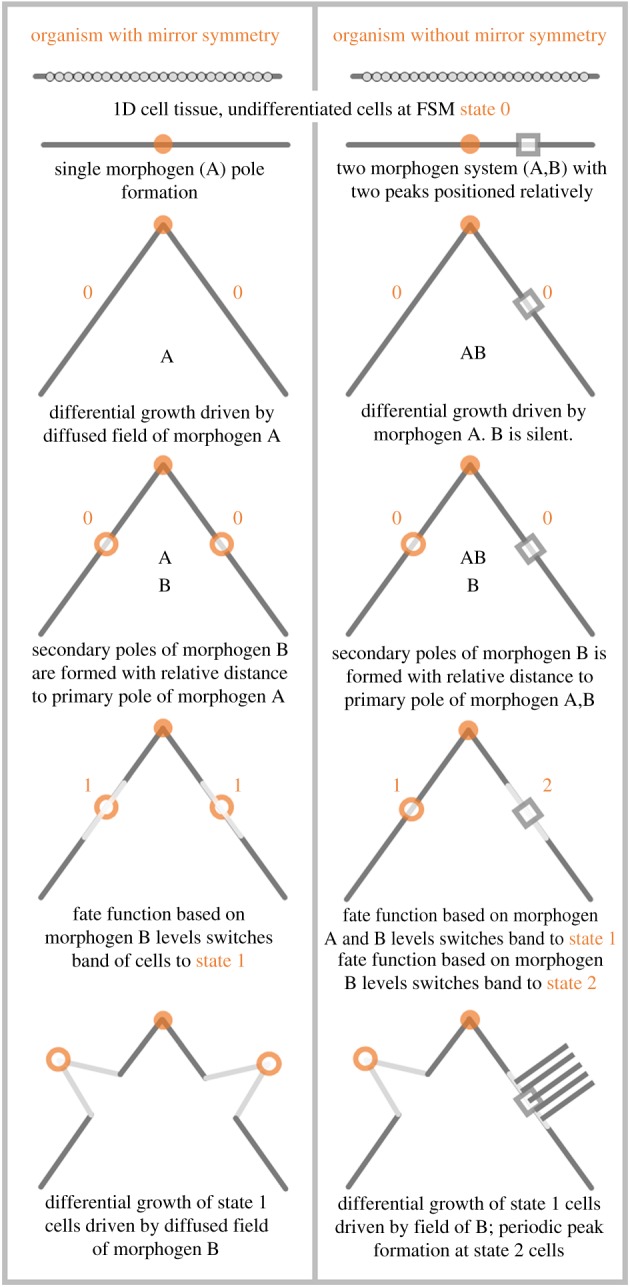
Sequential form making with FSM. Left and right columns depict the formation of organisms with and without mirror symmetry, respectively, on a 2D space, starting from the initial condition of undifferentiated linearly placed tissue cells. Left column results in arm–head–arm formation whereas the right column generates asymmetric beak–head–tail. (Online version in colour.)

Figure 4 illustrates the sequential form making with the FSM model. For illustration purposes, a 1D tissue with cells arranged on a line segment are depicted. Initially, all cells are at state 0. The chart illustrates the formation of an organism with mirror symmetry, and another organism with no symmetry axis in one dimension, in columns left and right, respectively.

In the left column, single symmetry breaking VRDD is used with one morphogen type ‘A’, resulting in a single pole with mirror symmetry. Growth is driven by a diffused field of such a pole (head formation). Secondary poles of morphogen type ‘B’ are positioned at a specified distance to primary pole ‘A’, based on the weights of interaction matrix **W**. The fate function, which uses the morphogen ‘B’ concentration level, creates two symmetric bands of tissue switching to state 1. Lastly, the two bands of state 1 have their subspatial growth driven by the diffused field of morphogen ‘B’ poles, resulting in a final form of arm–head–arm with mirror symmetry.

In the right column, double symmetry breaking VRDD is used with two morphogen types ‘A’,‘B’, having their respective source peaks as primary poles. Growth is driven by the diffused field of pole ‘A’ (head formation); ‘B’ is silent in terms of growth generation at this stage. The secondary pole of morphogen ‘B’ is positioned at specified distances from two primary poles, ‘A’ and ‘B’. The fate function that uses morphogen ‘A’ and ‘B’ concentration levels creates a band of tissue switching to state 1, asymmetric to the head (beak formation). The second fate function that uses the morphogen ‘B’ concentration level creates a band of tissue switching to state 2 (tail formation). Lastly, the cells in state 1 have their subspatial growth driven by the diffused field of morphogen ‘B’ poles, whereas the band of cells in state 2 execute a system with periodic peak formation, resulting in a final form of beak–head–tail with no mirror symmetry.

Hydra is an interesting model organism for morphogenetic studies because (i) its body plan is simple with a single axis from the head (hypostome and tentacles) to the glandular foot; and (ii) it continuously renews its body cells from migrating stem cells based on ever-available morphogenetic gradients for positional information; the hydra is therefore ‘an immortal and perpetual embryo’ [50]. Hydra can reproduce sexually with a few eggs produced in weeks, or, if well fed, it can reproduce by budding every 1.5–2 days, giving cloned offspring. Hydra has been extensively studied with transplantation experiments due its regeneration capability and continual axial morphogenetic gradients. In our hydra model, we modelled the embryonic development from a single egg; not the budding process for reproduction.

**Figure 5.**
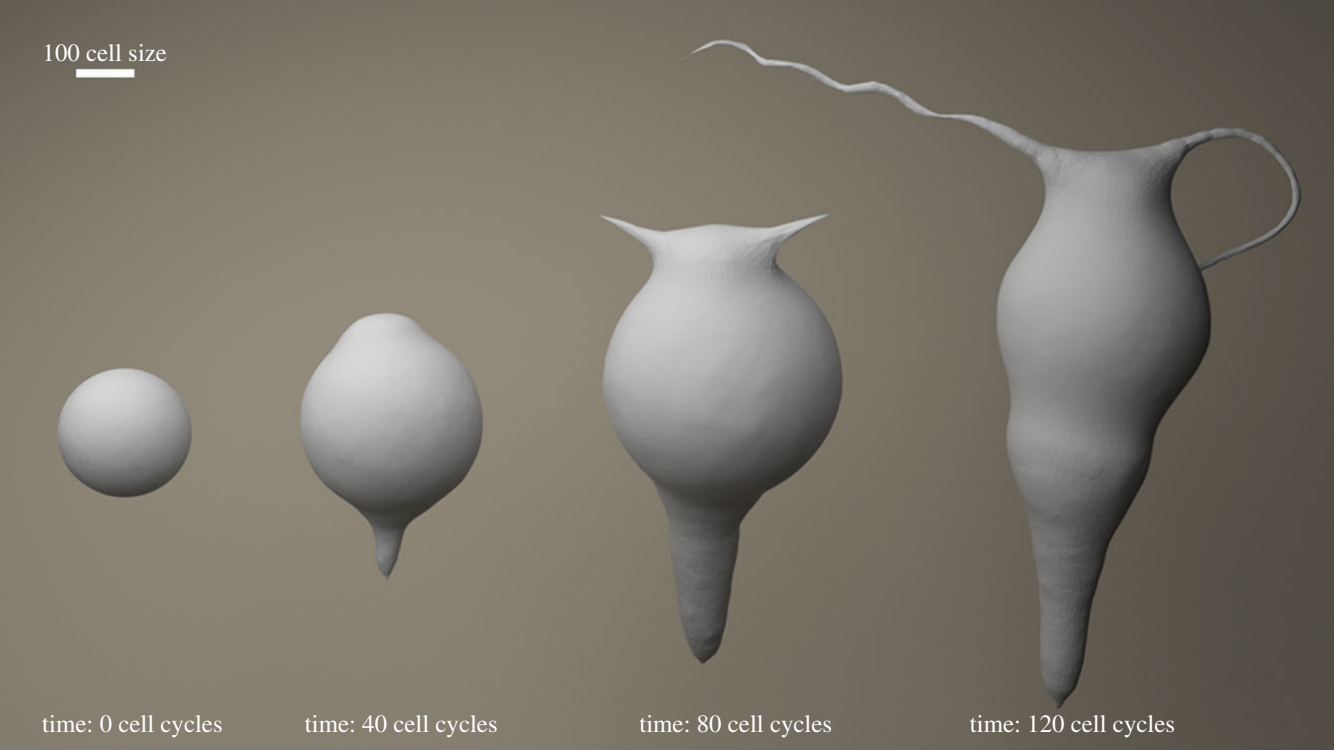
Hydra embryogenesis stages. Seven-state FSM with four morphogens are used to generate the hydra. Initial condition is a blastula with *ca* 2000 cells. After a 40 cell-cycle simulation time the anterior–posterior axis is formed using two-morphogen VRDD (R,G), followed by the head and tail region cell-state switching. A secondary pole formation at the ‘head’ subspatial domain is achieved by a separte two-morphogen (B,Y) VRDD running on cells with the ‘head polarize’ state. Tentacle growth results from a narrow band of cells switching to the ‘tentacle growth’ state, triggered by the fate function of B,Y morphogen concentration that defined the tentacle poles. The final form of the embryo is achieved in 120 cell cycles of simulation time with *ca* 8000 resultant cells on the topological surface. (Online version in colour.)

Using the sequential form-making technique, we generated a toy organism in the shape of a hydra, albeit with two tentacles for demonstration purposes. We implemented a seven-state FSM that consists of five developmental states, plus the omnipotent ground state and the final stasis state. The descriptions of states, including VRDD parameters, growth kernel and, most importantly, fate function with state transition connections, are provided in the electronic supplementary material. The simulation results of the developmental stages of our hydra are shown in figure 5.

### 4.3. Mutating a hydra

*In vivo* developmental mutants of a hydra have been produced by selective breeding [51] that had mini strains, maxi strains, multi-headed strains, nematocyst-deficient strains, regeneration-deficient strains and male sterile strains. These strains were transmitted to progeny by budding and cloned for developmental studies. It is challenging to generate transgenic mutant hydra; however, [52] and [53] reported a successful method for generating stable transgenic hydra lines which have since been used for signalling pathway studies. These studies showed that morphogenesis in hydra involves paracrine and juxtacrine signalling of Wnt and *β*-catenin [54], as well as Notch–Delta activity [55] for creation of tissue boundaries.

**Figure 6.**
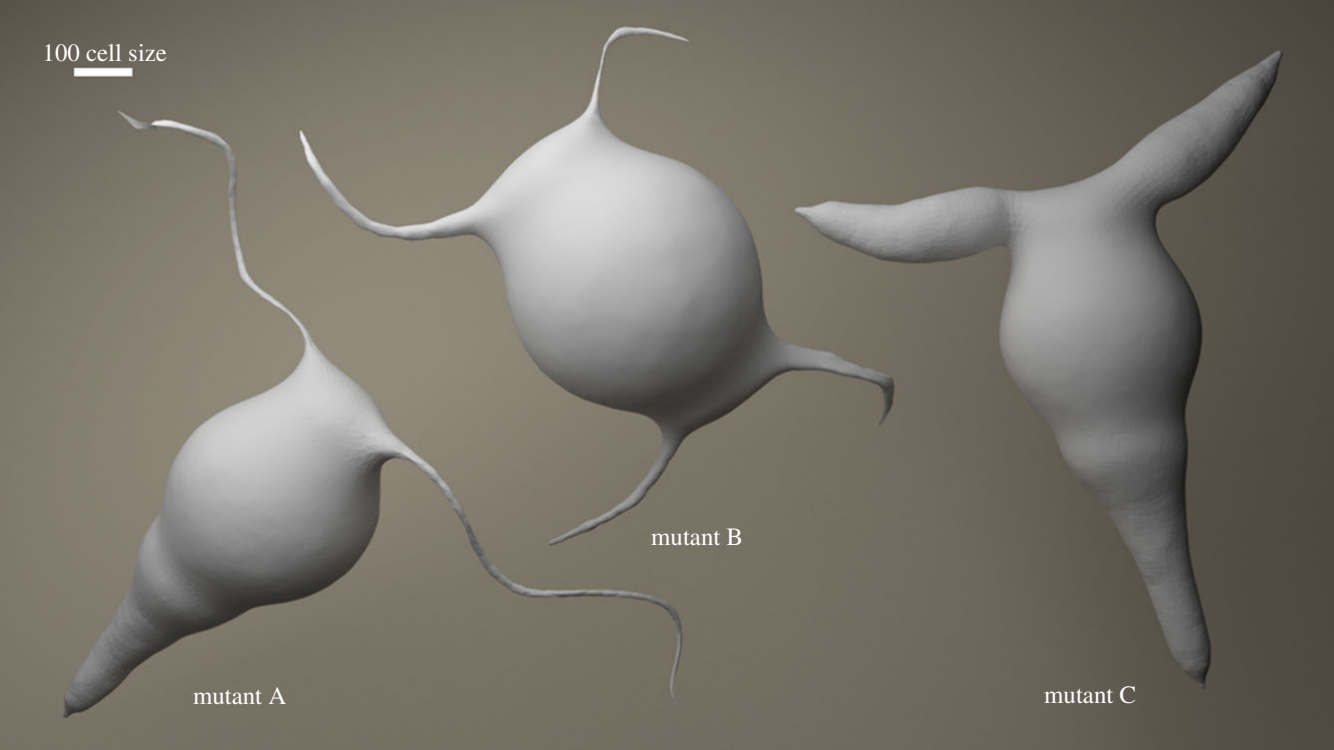
Hydra mutants. Mutants A and C are generated by varying growth kernel values as a type of continuous mutation. Mutant B is generated as a jump mutation where state transition of the FSM is rewired, leading to double head formation. (Online version in colour.)

In our continuous-mutation experiments, we varied some of the structural parameters of our synthetic genome, which resulted in fat hydra and long-tentacled hydra. These mutants are topologically homologous to the wild-type as shown in figure 6 (mutants A and C).

More interestingly our jump-mutation-type alterations of the genotype resulted in double head formation. In this experiment, cell-fate functions that differentiate cells to tail-type were rewired to switch to head-type in the synthetic genome, which resulted in stably generated mutants with two heads at opposite ends of the primary body axis as shown in figure 6 (mutant B). These *in silico* mutants are similar to two-headed mutants that were bred by Sugiyama & Fujisawa [51] and resemble homeotic mutants in *Drosophila* with legs emanating from the head [56]. Multi-headedness is also observed in the transgenic mutants that were produced by Wittlieb *et al*. [52]; however, these are not based on generation of two heads in axial opposites but rather generation of multi-heads throughout the body. We show that genotype to phenotype mapping is not necessarily continuous; a small alteration of FSM connectivity resulted in a radical change in phenotype. Random walk on genotype space with small variations can result in discontinuity on phenotype space. This may be an explanation of observed discontinuity in the fossil records.

### 4.4. Other organism forms

We experimented with generating various other organism forms. To streamline the design of genotypes for such forms, we developed a drag-and-drop design tool (DNAMaker). The FSM state table generated by DNAMaker was then injected into the embryogenesis simulation environment (EmbryoDeveloper). Some selected phenotypes at the end of embryonic development are provided in the electronic supplementary material.

## 5. Methods

All simulations were done using the code written by us in C++ using the standard library only. Visual Studio 2015 was used for code development. Mesh Class, which has mesh graph data structure as well as VRDD and FSM-related methods, is provided under mesh.h and mesh.cpp. Two main tools for genotype design and phenotype generation were also coded in C++ (DNAMaker and EmbryoDeveloper). For graphics output, we used the openFrameworks library (free and open source C++ toolkit) to visualize the organism in mesh form in real time. High-quality renderings for final visual outputs were done in Blender (free and open source 3D creation suite). All simulations that used partial differential equations of the VRDD system were implemented using the outlined discretization methods.

Simulations were run on an Intel Core i7 CPU of a DELL XPS laptop with 16 GB of RAM and a 64-bit Windows operating system. Real-time graphic output and high-resolution rendering were done using an Nvidia GeForce GTX 1050 GPU. Simulations of embryonic development starting with *ca* 10 000 cells of blastula stage, ending up with *ca* 100 000 cells of final form, took *ca* 5 min.

## 6. Discussion

In this paper, we introduced the VRDD system as a novel concept which can generate bodyplans of fundamental forms by self-organization. We then elaborated on an FSM model of the genetic regulatory network that uses our VRDD model. The result of VRDD combined with the FSM model is spatial cell differentiation during embryogenesis that can be used for hierarchical modelling of complicated forms. We have demonstrated that our concepts are capable of generating self-organized bodyplans from which we developed life-like organism forms *in silico*.

Our current implementations generate forms of lower animals such as hydra or jellyfish. However, the VRDD model is capable of generating dual-axis bodyplans (anterior–posterior and dorsoventral) of vertebrates by self-organization. An important next step in our work will be to investigate dynamics that can robustly generate a given number of segments (such as five fingers) and anatomical ratios (such as limb length proportions). We are cognizant of the fact that the physical layer of our simulation model, which is currently based on elastic membrane, needs to be expanded to incorporate aggregation dynamics for hard tissue formation.

There are several observations worthy of further study, including the analytical treatment of VRDD attractor dynamics (basins of attraction, bifurcation conditions and convergence rates), field profile of a standalone morphogen versus coupled systems and morphogen reuse in spatial hierarchical subdomains.

We note that the Four Colour Theorem in mathematics says that any partitioning of a topologically spheric surface can be coloured with four colours without two neighbouring patches having the same colour. We speculate that this would lead to a lower bound in the number of morphogen types that can be used in generating any arbitrary form using an FSM model with the morphogen reuse in the spatial subdomains. Referring back to figure 4, we have outlined the conceptual framework for defining spatial subdomains in 1D tissue with two morphogens using VRDD dynamics. When we expand the concept to 2D tissue, the topological dimensionality of the problem is increased by twofold. Hence we believe that four morphogens could be sufficient to specify any spatial subdomain in two dimensions with the same approach as we described in one dimension. Linking this to the Four Colour Theorem is clearly not trivial as the proof of the Four Colour Theorem itself had been a long-standing challenge in mathematics which was completed in 1976 as a computer-assisted proof [57]. A key challenge to our conjecture would be that organisms are not two dimensional, but three. We would claim that embryonic growth is fundamentally a 2D phenomenon where tissue is layered as 2D shells. We acknowledge that linking the Four Colour Theorem to 2D tissue growth using four fundamental morphogens is a highly speculative proposition that will require significant further theoretical work.

Our concepts provide possible mechanisms for spatially distributed nonlinear dynamical systems for embryogenesis, which have so far been restricted to Turing-like pattern formation systems [1] or the ‘positional information’ theory of development by Wolpert [49]. Whether life really uses concepts similar to the ones we have developed remains to be investigated. Notably, our concepts are capable of generating life-like forms robustly and efficiently, which are desirable features for natural selection. Our work is conceptual, yet it can lead to practical applications in the emerging fields of morphogenetic engineering, soft robotics and biomimetic architectural design.

## Supplementary Material

Hydra Genome Code

## Supplementary Material

Other Forms Generated

## Supplementary Material

Jellyfish Development

## Supplementary Material

Periodic Tentacle Development

## Supplementary Material

Single Axis Bodyplan Development

## Data accessibility

The code for reproducing the simulation results is available from https://github.com/Merkurt68/DNAMaker, https://github.com/Merkurt68/EmbryoDeveloper.

## References

[1] Turing AM 1952 The chemical basis of morphogenesis. Phil. Trans. R. Soc. Lond. B 237, 37–72. (10.1098/rstb.1952.0012) PMC4360114 25750229

[2] Gierer A, Meinhardt H 1972 A theory of biological pattern formation. Kybernetik 12, 30–39. (10.1007/BF00289234) 4663624

[3] Kondo S, Miura T 2010 Reaction-diffusion model as a framework for understanding biological pattern formation. Science 329, 1616–1620. (10.1126/science.1179047) 20929839

[4] Marcon L, Sharpe J 2012 Turing patterns in development: what about the horse part? Curr. Opin. Genet. Dev. 22, 578–584. (10.1016/j.gde.2012.11.013) 23276682

[5] Wolpert L 1981 Positional information and pattern-formation. Phil. Trans. R. Soc. Lond. B 295, 441–450. (10.1098/rstb.1981.0152) 6117904

[6] Rogers KW, Schier AF 2011 Morphogen gradients: from generation to interpretation. Annu. Rev. Cell Dev. Biol. 27, 377–407. (10.1146/annurev-cellbio-092910-154148) 21801015

[7] Summerbell D, Wolpert L 1973 Precision of development in chick limb morphogenesis. Nature 244, 228–230. (10.1038/244228a0) 4583096

[8] French V, Bryant PJ, Bryant SV 1976 Pattern regulation in epimorphic fields. Science 193, 969–981. (10.1126/science.948762) 948762

[9] Entchev EV, Schwabedissen A, Gonzalez-Gaitan M 2000 Gradient formation of the TGF-β homolog Dpp. Cell 103, 981–991. (10.1016/S0092-8674(00)00200-2) 11136982

[10] Teleman AA, Cohen SM 2000 Dpp gradient formation in the *Drosophila* wing imaginal disc. Cell 103, 971–980. (10.1016/S0092-8674(00)00199-9) 11136981

[11] Ingham PW, McMahon AP 2001 Hedgehog signaling in animal development: paradigms and principles. Gene. Dev. 15, 3059–3087. (10.1101/gad.938601) 11731473

[12] Stamataki D, Ulloa F, Tsoni SV, Mynett A, Briscoe J 2005 A gradient of Gli activity mediates graded Sonic Hedgehog signaling in the neural tube. Gene. Dev. 19, 626–641. (10.1101/gad.325905) 15741323 PMC551582

[13] Chamberlain CE, Jeong J, Guo C, Allen BL, McMahon AP 2008 Notochord-derived Shh concentrates in close association with the apically positioned basal body in neural target cells and forms a dynamic gradient during neural patterning. Development 135, 1097–1106. (10.1242/dev.013086) 18272593

[14] Balaskas N, Ribeiro A, Panovska J, Dessaud E, Sasai N, Page KM, Briscoe J, Ribes V 2012 Gene regulatory logic for reading the sonic hedgehog signaling gradient in the vertebrate neural tube. Cell 148, 273–284. (10.1016/j.cell.2011.10.047) 22265416 PMC3267043

[15] Wolpert L 2011 Positional information and patterning revisited. J. Theor. Biol. 269, 359–365. (10.1016/j.jtbi.2010.10.034) 21044633

[16] Lander AD 2007 Morpheus unbound: reimagining the morphogen gradient. Cell 128, 245–256. (10.1016/j.cell.2007.01.004) 17254964

[17] Kerszberg M, Wolpert L 1998 Mechanisms for positional signalling by morphogen transport: a theoretical study. J. Theor. Biol. 191, 103–114. (10.1006/jtbi.1997.0575) 9593661

[18] Ibanes M, Belmonte JCI 2008 Theoretical and experimental approaches to understand morphogen gradients. Mol. Syst. Biol. 4, 176 (10.1038/msb.2008.14) 18364710 PMC2290935

[19] Kerszberg M, Wolpert L 2007 Specifying positional information in the embryo: looking beyond morphogens. Cell 130, 205–209. (10.1016/j.cell.2007.06.038) 17662932

[20] Eldar A, Rosin D, Shilo BZ, Barkai N 2003 Self-enhanced ligand degradation underlies robustness of morphogen gradients. Dev. Cell 5, 635–646. (10.1016/S1534-5807(03)00292-2) 14536064

[21] Eldar A, Shilo BZ, Barkai N 2004 Elucidating mechanisms underlying robustness of morphogen gradients. Curr. Opin. Genet. Dev. 14, 435–439. (10.1016/j.gde.2004.06.009) 15261661

[22] Green JBA, Sharpe J 2015 Positional information and reaction-diffusion: two big ideas in developmental biology combine. Development 142, 1203–1211. (10.1242/dev.114991) 25804733

[23] Meinhardt H 1986 Formation of symmetrical and asymmetric structures during development of higher organisms. Comput. Math. Appl. B 12, 419–433. (10.1016/0898-1221(86)90163-X)

[24] Meinhardt H 1992 Pattern-formation in biology—a comparison of models and experiments. Rep. Prog. Phys. 55, 797–849. (10.1088/0034-4885/55/6/003)

[25] Meinhardt H 1989 Tailoring and coupling of reaction-diffusion systems to obtain reproducible complex pattern-formation during development of the higher organisms. Appl. Math. Comput. 32, 103–135. (10.1016/0096-3003(89)90090-8)

[26] Meinhardt H 1995 Development of higher organisms—how to avoid error propagation and chaos. Physica D 86, 96–103. (10.1016/0167-2789(95)00091-H)

[27] Meinhardt H 2001 Organizer and axes formation as a self-organizing process. Int. J. Dev. Biol. 45, 177–188. 11291845

[28] Meinhardt H 2009 Models for the generation and interpretation of gradients. Cold Spring Harb. Perspect. Biol. 1, a001362 (10.1101/cshperspect.a001362) 20066097 PMC2773622

[29] Meinhardt H 2000 Models for organizer and notochord formation. Cr. Acad. Sci. Iii-Vie 323, 23–30. (10.1016/S0764-4469(00)00104-9) 10742908

[30] Wolpert L, Arias AM, Tickle C 2015 Principles of development. Oxford, UK: Oxford University Press.

[31] Wieschaus E 2016 Positional information and cell fate determination in the early Drosophila embryo. Curr. Top. Dev. Biol. 117, 567–579. (10.1016/bs.ctdb.2015.11.020) 26970001

[32] Muller P, Rogers KW, Yu SZR, Brand M, Schier AF 2013 Morphogen transport. Development 140, 1621–1638. (10.1242/dev.083519) 23533171 PMC3621481

[33] Vasilopoulos G, Painter KJ 2016 Pattern formation in discrete cell tissues under long range filopodia-based direct cell to cell contact. Math. Biosci. 273, 1–15. (10.1016/j.mbs.2015.12.008) 26748293

[34] Kauffman S 1971 Gene regulation networks: a theory for their global structure and behaviors. Curr. Top. Dev. Biol. 6, 145–182. (10.1016/S0070-2153(08)60640-7) 5005757

[35] Ruz GA, Goles E, Montalva M, Fogel GB 2014 Dynamical and topological robustness of the mammalian cell cycle network: a reverse engineering approach. Biosystems 115, 23–32. (10.1016/j.biosystems.2013.10.007) 24212100

[36] Kaneko K, Yomo T 1994 Cell-division, differentiation and dynamic clustering. Physica D 75, 89–102. (10.1016/0167-2789(94)90277-1)

[37] Mjolsness E, Sharp DH, Reinitz J 1991 A connectionist model of development. J. Theor. Biol. 152, 429–453. (10.1016/S0022-5193(05)80391-1) 1758194

[38] Salazar-Ciudad I, Garcia-Fernandez J, Sole RV 2000 Gene networks capable of pattern formation: from induction to reaction-diffusion. J. Theor. Biol. 205, 587–603. (10.1006/jtbi.2000.2092) 10931754

[39] Hengenius JB, Gribskov M, Rundell AE, Fowlkes CC, Umulis DM 2011 Analysis of gap gene regulation in a 3D organism-scale model of the *Drosophila melanogaster* embryo. PLoS ONE 6, e26797 (10.1371/journal.pone.0026797) 22110594 PMC3217930

[40] Salazar-Ciudad I 2009 Looking at the origin of phenotypic variation from pattern formation gene networks. J. Biosci. 34, 573–587. (10.1007/s12038-009-0075-6) 19920342

[41] Fisher AG 2002 Cellular identity and lineage choice. Nat. Rev. Immunol. 2, 977–982. (10.1038/nri958) 12461570

[42] Jaeger J, Reinitz J 2006 On the dynamic nature of positional information. Bioessays 28, 1102–1111. (10.1002/bies.20494) 17041900

[43] Allena R, Munoz JJ, Aubry D 2013 Diffusion-reaction model for *Drosophila* embryo development. Comput. Method. Biomec. 16, 235–248. (10.1080/10255842.2011.616944) 21970322

[44] Mercker M, Brinkmann F, Marciniak-Czochra A, Richter T 2016 Beyond Turing: mechanochemical pattern formation in biological tissues. Biol. Direct 11, 235 (10.1186/s13062-016-0124-7) PMC4857296 27145826

[45] Delile J, Herrmann M, Peyrieras N, Doursat R 2017 A cell-based computational model of early embryogenesis coupling mechanical behaviour and gene regulation. Nat. Commun. 8, 13929 (10.1038/ncomms13929) 28112150 PMC5264012

[46] Pascalie J, Potier M, Kowaliw T, Giavitto J-L, Michel O, Spicher A, Doursat R 2016 Developmental design of synthetic bacterial architectures by morphogenetic engineering. ACS Synth. Biol. 5, 842–861. (10.1021/acssynbio.5b00246) 27244532

[47] Izaguirre JA *et al.* 2004 CompuCell, a multi-model framework for simulation of morphogenesis. Bioinformatics 20, 1129–1137. (10.1093/bioinformatics/bth050) 14764549

[48] Cickovski TM, Huang CB, Chaturvedi R, Glimm T, Hentschel HGE, Alber MS, Glazier JA, Newman SA, Izaguirre JA 2005 A framework for three-dimensional simulation of morphogenesis. IEEE ACM Trans. Comput. Bioinform. 2, 273–288. (10.1109/tcbb.2005.46) 17044166

[49] Wolpert L 2016 Positional information and pattern formation. Curr. Top. Dev. Biol. 117, 597–608. (10.1016/bs.ctdb.2015.11.008) 26970003

[50] Muller WA 1996 Head formation at the basal end and mirror-image pattern duplication in Hydra vulgaris. Int. J. Dev. Biol 40, 1119–1131. 9032017

[51] Sugiyama T, Fujisawa T 1977 Genetic-analysis of developmental mechanisms in Hydra. 1. Sexual reproduction of *Hydra magnipapillata* and isolation of mutants. Dev. Growth Differ. 19, 187–200. (10.1111/j.1440-169X.1977.00187.x) 37280974

[52] Wittlieb J, Khalturin K, Lohmann JU, Anton-Erxleben F, Bosch TCG 2006 Transgenic Hydra allow in vivo tracking of individual stem cells during morphogenesis. Proc. Natl Acad. Sci. USA 103, 6208–6211. (10.1073/pnas.0510163103) 16556723 PMC1458856

[53] Khalturin K, Anton-Erxleben F, Milde S, Plötz C, Wittlieb J, Hemmrich G, Bosch TCG 2007 Transgenic stem cells in Hydra reveal an early evolutionary origin for key elements controlling self-renewal and differentiation. Dev. Biol. 309, 32–44. (10.1016/j.ydbio.2007.06.013) 17659272

[54] Lengfeld T *et al.* 2009 Multiple Wnts are involved in Hydra organizer formation and regeneration. Dev. Biol. 330, 186–199. (10.1016/j.ydbio.2009.02.004) 19217898

[55] Munder S *et al.* 2013 Notch-signalling is required for head regeneration and tentacle patterning in Hydra. Dev. Biol. 383, 146–157. (10.1016/j.ydbio.2013.08.022) 24012879

[56] Nussleinvolhard C, Wieschaus E 1980 Mutations affecting segment number and polarity in Drosophila. Nature 287, 795–801. (10.1038/287795a0) 6776413

[57] Appel KI, Haken W 1989 Every planar map is four colorable. Contemp. Math. 98, 741 (10.1090/conm/098)

