# Supplementary material for: Emergence of form in embryogenesis
Source: J R Soc Interface. 2018 Nov 14;15(148):20180454. doi: 10.1098/rsif.2018.0454 (PMC6283983; doi:10.1098/rsif.2018.0454)
Supplement: Hydra Genome Code [file rsif20180454supp1.pdf]

State	Description	VRDD	Growth	Fate	State+
0	Blastula growth	--	u	T>	1
1	Blastula polarize	<RG>	--	T>	x
				R>	2
				G>	3
2	Tail growth	--	u+r	T>	x
3	Head growth	--	u+g	T>	4
4	Head polarize	<BY>	--	T>	x
				B>||G>	5
5	Tentacle growth	--	u+b+y	T>	x
x	Stasis	--	--	--	--

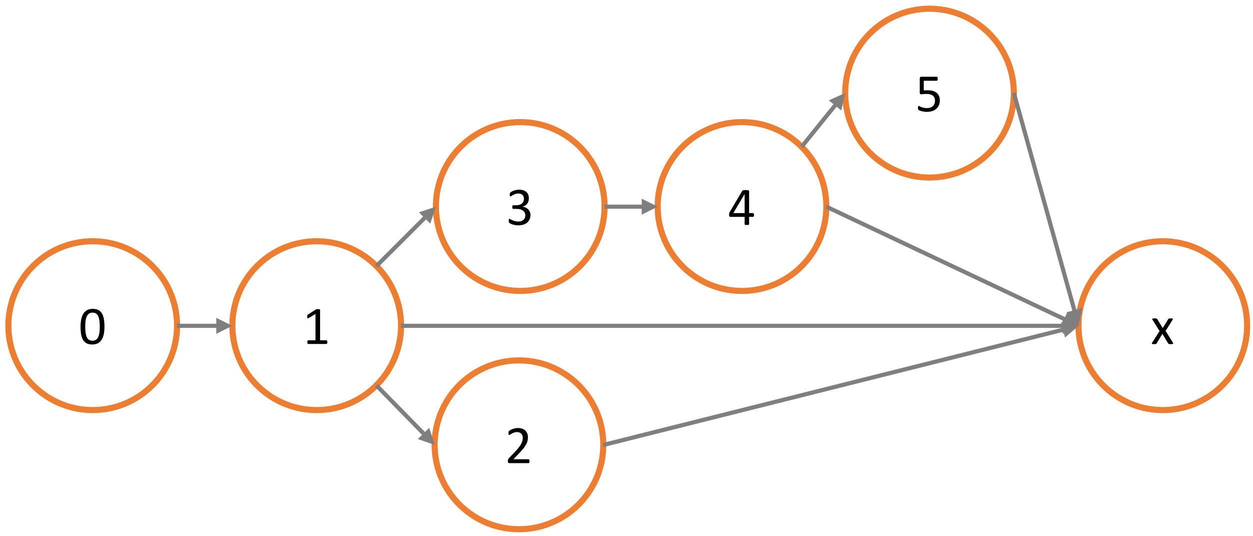

Mutation type	State	Alteration	Result
Continuous	5	$u \rightarrow u^{++}$	Fat tentacles
Continuous	5	$b,y \rightarrow b^{++},y^{++}$	Long tentacles
Jump	1	R>: 2 → 3	Double head
