# Supplementary material for: Emergence of form in embryogenesis
Source: J R Soc Interface. 2018 Nov 14;15(148):20180454. doi: 10.1098/rsif.2018.0454 (PMC6283983; doi:10.1098/rsif.2018.0454)
Supplement: Other Forms Generated [file rsif20180454supp2.pdf]

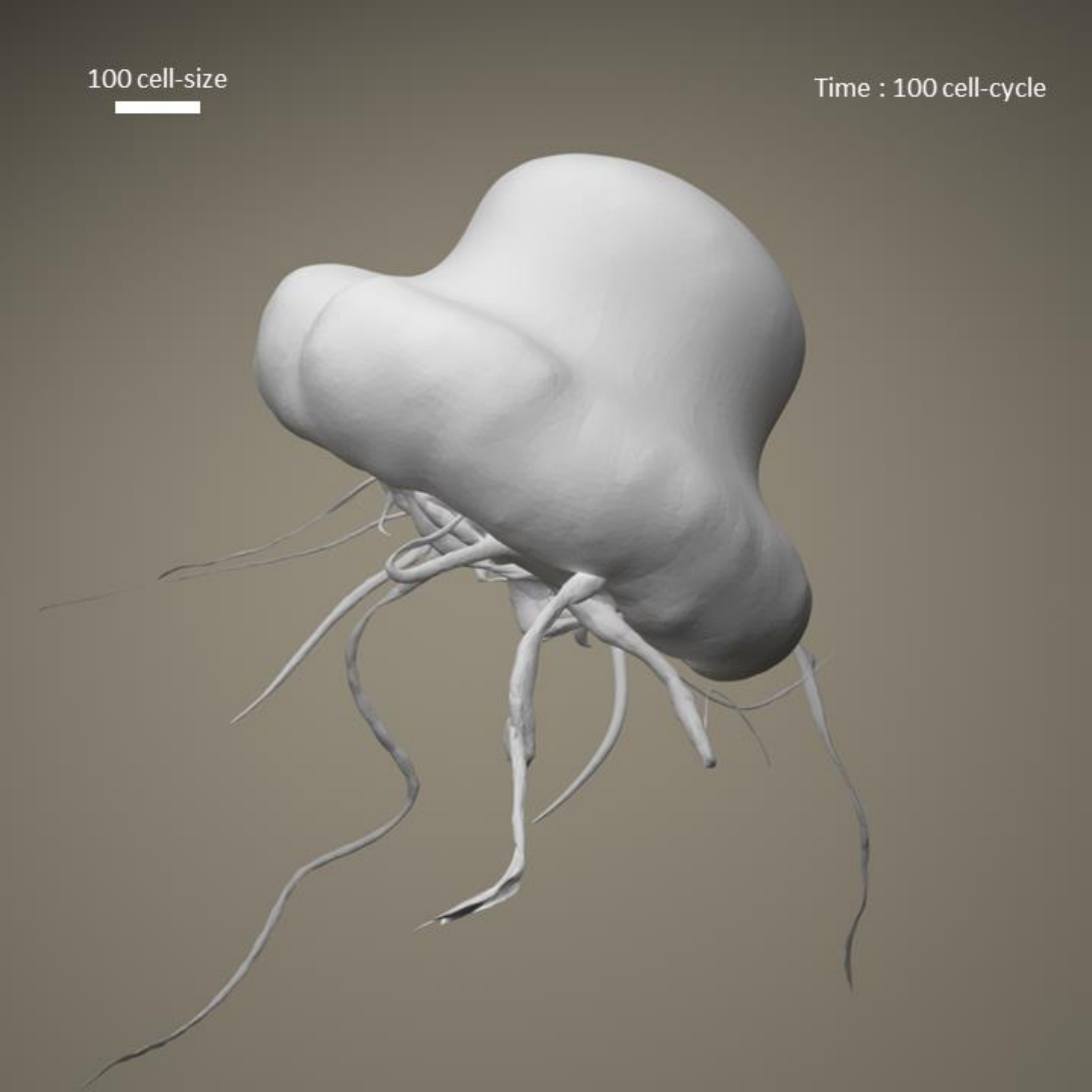

100 cell-size
Time : 100 cell-cycle

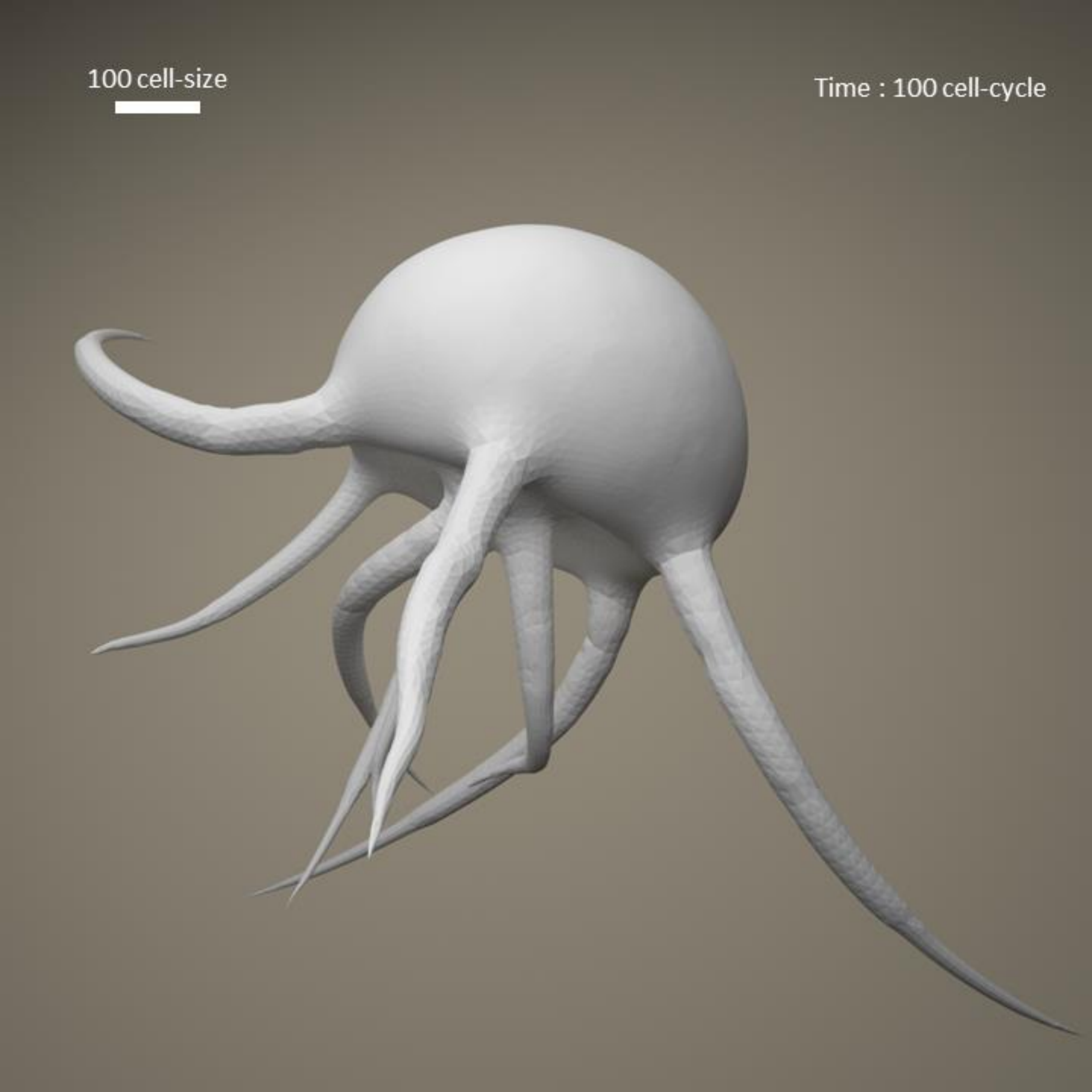
100 cell-size
Time : 100 cell-cycle

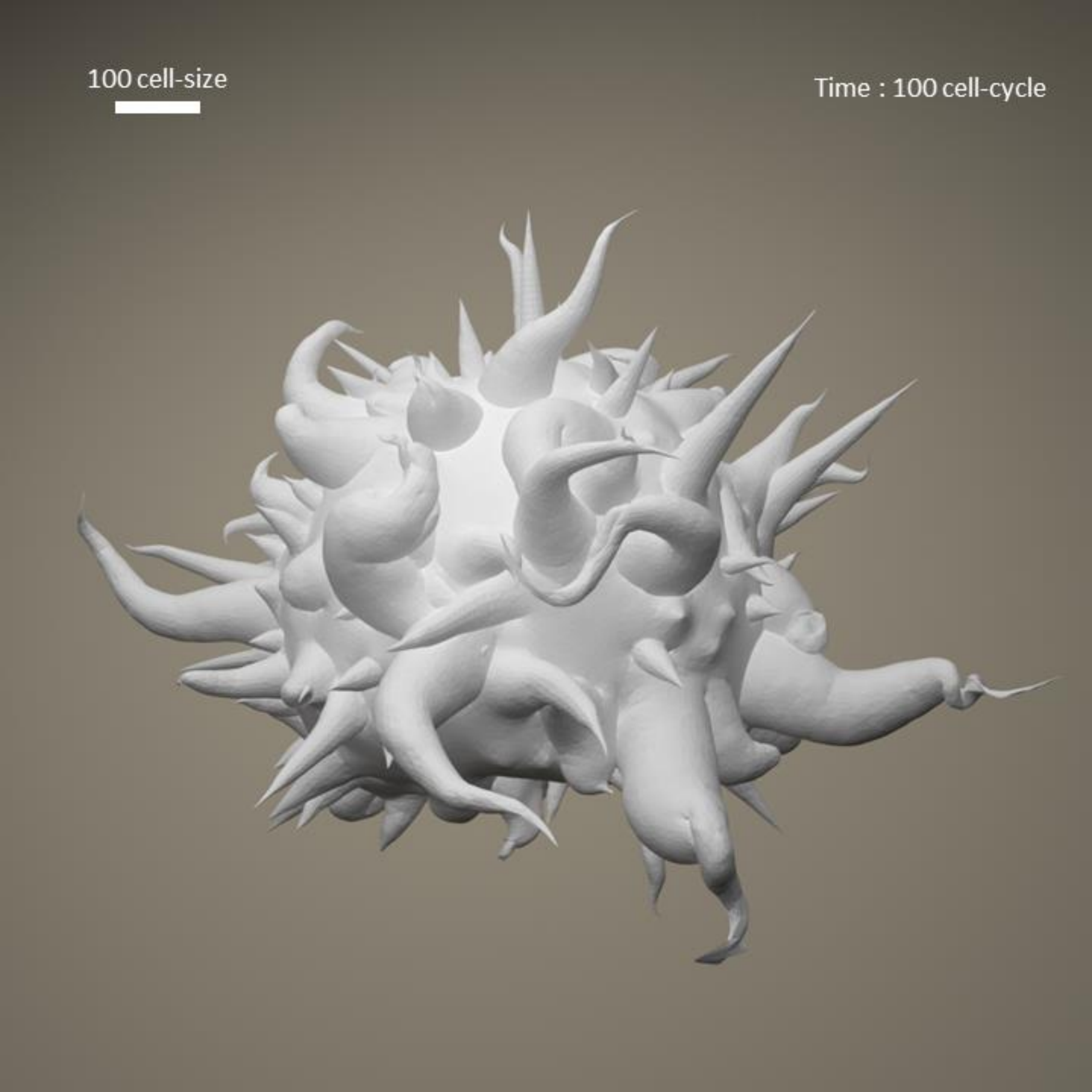
100 cell-size
Time : 100 cell-cycle

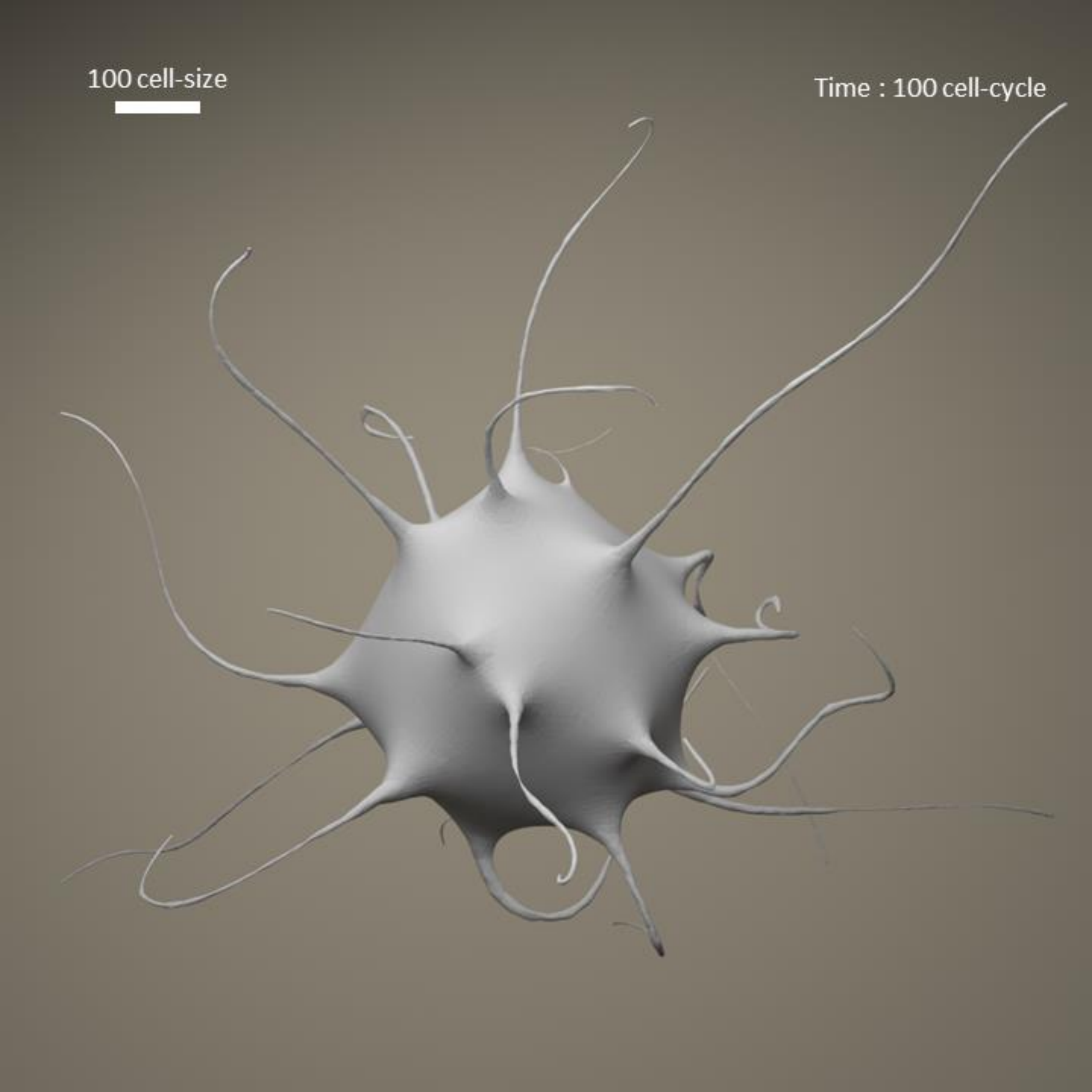
100 cell-size
Time : 100 cell-cycle
